# Transcriptome and Expression Analysis of Glycerol Biosynthesis-Related Genes in *Glenea cantor* Fabricius (Cerambycidae: Lamiinae)

**DOI:** 10.3390/ijms252111834

**Published:** 2024-11-04

**Authors:** Taihui Lan, Ranran Su, Zishu Dong, Xin Tong, Xialin Zheng, Xiaoyun Wang

**Affiliations:** 1 Guangxi Key Laboratory of Agric-Environment and Agric-Products Safety, College of Agriculture, Guangxi University, Nanning 530004, China; lth5371@163.com (T.L.); srr18838933489@126.com (R.S.); tongxin@gxu.edu.cn (X.T.); zheng-xia-lin@163.com (X.Z.); 2 Institute of Advanced Study, Jiangxi University of Chinese Medicine, Nanchang 330004, China; dongzishu1668@163.com

**Keywords:** longhorn beetles, full-length transcriptome, cold hardiness, phylogenetic tree, expression patterns

## Abstract

*Glenea cantor* Fabricius (Cerambycidae: Lamiinae) is an important pest that damages kapok trees in Southeast Asia with a wide adaptability to temperature. Glycerol is a protectant and energy source for insects in low-temperature environments. However, glycerol biosynthesis-related genes at the molecular level are limited in *G. cantor*. In this study, the supercooling points and freezing points at different stages were measured, and the cold hardiness of male and female pupae significantly differed. Moreover, a full-length transcriptome of *G. cantor* was established; glycerol kinase (GK) and glycerol-3-phosphate dehydrogenase (GPDH) genes, which are related to glycerol metabolism, were identified, with a special focus on their expression profiles. A total of 24,476 isoforms stemmed from the full-length transcriptome, along with 568 lncRNAs, 56 transcription factor (TF) families, and 1467 alternative splicing (AS) events. The KEGG pathway enrichment analysis revealed that the isoforms associated with AS were enriched primarily in glycerolipid and glycerophospholipid metabolism. In total, three GK genes and one GPDH gene were identified, and GcGK1 and GcGK3 presented differential sex expression during the pupal stage, which may play a role in thermal adaptability. This study provides a valuable transcriptional database of *G. cantor* and helps to elucidate the function of glycerol in the thermal adaptation mechanism of longhorn beetles.

## 1. Introduction

Global warming leads to geographic variation in species, as well as morphological and biochemical responses [1,2], making insect temperature adaptability a hot topic. The arrival of winter or sudden cooling frequently affects insect diapause and reproduction [3,4]. Many insects cope with such adversity in various ways. In addition to relying on inorganic ions to coordinate physical balance, they can also generate specific protective compounds to prevent internal tissue from freezing [5,6]. As energy sources, polyols and glycogen are not only beneficial for the biological protection of various insects facing long-term exposure to low-temperature environments but they also accumulate, causing an increase in hemolymph osmolality in *Anoplophora glabripennis* [7,8]. Moreover, glycerol plays an important role in the cold resistance mechanism of insects, which allows *Bactrocera dorsalis* to adapt to cold [9] and acts as a cryoprotectant in *Hippodamia variegata* [10]. Therefore, the characterization of glycerol-3-phosphate dehydrogenase (GPDH) and glycerol kinase (GK), two highly important parts of the glycerol metabolism system, is conducive to discerning the mechanism of cold resistance adaptability, especially during overwintering. In glycerol oxidative degradation metabolism, the product that is catalyzed by glycerol kinase, glycerol-3-phosphate, is subsequently oxidized and dehydrogenated by GPDH to produce dihydroxyacetone phosphate, which is involved in the tricarboxylic acid cycle or gluconeogenesis. However, glycerol synthesis metabolism is a reversible process [11].

The regulation of gene expression is a decisive scientific topic in the field of molecular biology. In the past decade, RNA sequencing (RNA-seq) technology has been widely applied, and the emergence of Pacific Biosciences sequencing (PacBio-seq) has promoted transcriptomic research in various organisms. Currently, single-molecule real-time sequencing (SMRT-seq) yields high-quality full-length (FL) transcripts that are often used in conjunction with RNA-seq, increasing the number of insect genome annotations and functional studies [12,13]. Bioinformatics analysis, including alternative splicing (AS), transcription factor (TF), and long noncoding RNA (lncRNA) analyses, can be used to predict, on the basis of transcriptome data, different meaningful parts that jointly enter the cascade reaction program of gene expression [14,15,16]. SMRT-Seq has an advantage in yielding FL transcripts, especially for non-model organisms without a reference genome [17].

*Glenea cantor* (Fabricius), which prefers tender kapok trees, is widely spread throughout Southeast Asia and southern China [18]. The insect produces four generations per year and does not overwinter or diapause under suitable temperatures and host conditions. When the temperature increases in the winter, young larvae still resume their feeding activities [19]. For *G. cantor*, the antennal and leg transcriptomes have been identified [20,21], and understanding of the olfactory system with sex differences has increased. However, the molecular resources and glycerol metabolism system of *G. cantor* are unknown.

In this study, full-length SMRT transcriptome analysis was conducted to identify valuable basic physiological resources for *G. cantor*. Moreover, the transcription levels and evolutionary relationships of GcGKs and GcGPDH were detected. Our findings provide new insights into glycerol metabolism in *G. cantor*, which could be used as a reference for temperature adaptability research.

## 2. Results

### 2.1. Supercooling and Freezing Points at Different Stages of G. cantor Development

The supercooling points and freezing points of mature larvae, pupae, and adults of *G. cantor* were measured using an intelligent insect supercooling point tester and supporting software, and the differences in the supercooling points and freezing points of different insect states were compared. The results revealed that the average supercooling point of female pupae was the lowest, at −22.16 ± 1.23 °C. Second, the average supercooling point of male pupae was lower than that of the other three insect stages except for female pupae. The average freezing point of mature larvae was the highest, at −9.20 ±1.14 °C. Pupae have strong cold resistance, but mature larvae and adults have weak cold resistance (Table 1).

### 2.2. Outcome of Full-Length Transcriptome Sequencing

A total of 9,716,761 subreads with an average length of 2311 bp were generated from pooled RNA via the PacBio Sequel II platform (Table 2). All subreads were corrected and merged to yield 363,474 circular consensus sequences (CCSs). The full-length nonchimeric (FLNC) reads resulted in 24,476 high-quality nonredundant transcripts with a mean length of 2686 bp for subsequent analysis. Among them, the N50 was 3030 bp, and the GC content was 44.18%, indicating the reliability and completeness of the SMRT data. The raw data of *G. cantor* have been submitted to the NCBI with access number PRJNA1107111.

### 2.3. Transcriptome Functional Annotation

**Figure 1:**
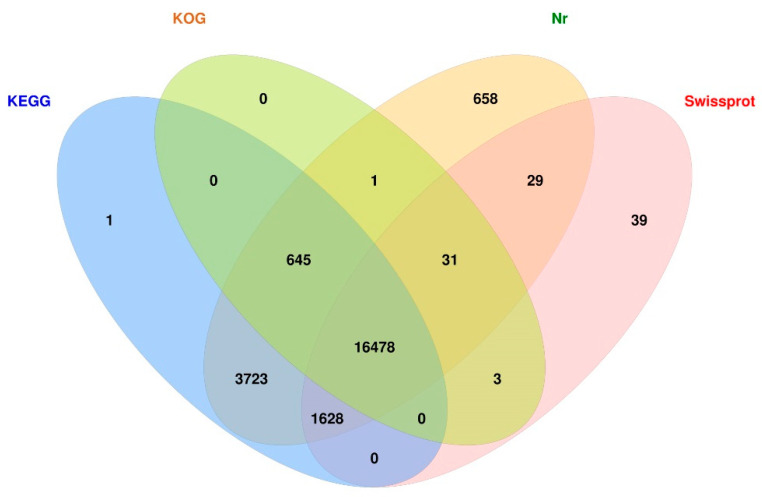
Venn diagram showing the statistical annotation results of the four public databases.

To reveal an elementary function of the full-length transcriptome of *G. cantor*, we annotated 24,476 isoforms in four public databases. The Venn diagram clearly shows that 23,236 isoforms were annotated by at least one database and that 16,478 isoforms could be simultaneously annotated in the four aforementioned databases (Figure 1).

The isoforms of *G. cantor* that may participate in metabolic pathways were combined with the KEGG database. Notably, among the top 10 annotated terms, the most enriched category was related to metabolic pathways (Table S2).

**Figure 2:**
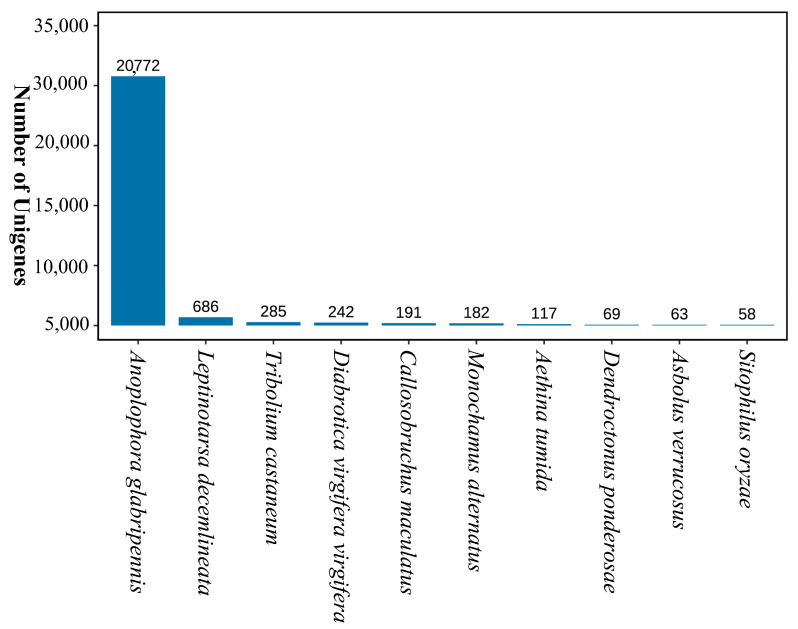
Bar plot showing homologous species distribution of transcripts in the Nr database.

In the Nr database, 20,772 homologous transcripts (89.56%) matched *A. glabripennis* (Figure 2). Additionally, *Leptinotarsa decemlineata*, *Tribolium castaneum,* and *Diabrotica virgifera virgifea* presented the highest similarity with the remaining three species.

### 2.4. LncRNAs and TFs in G. cantor

**Figure 3:**
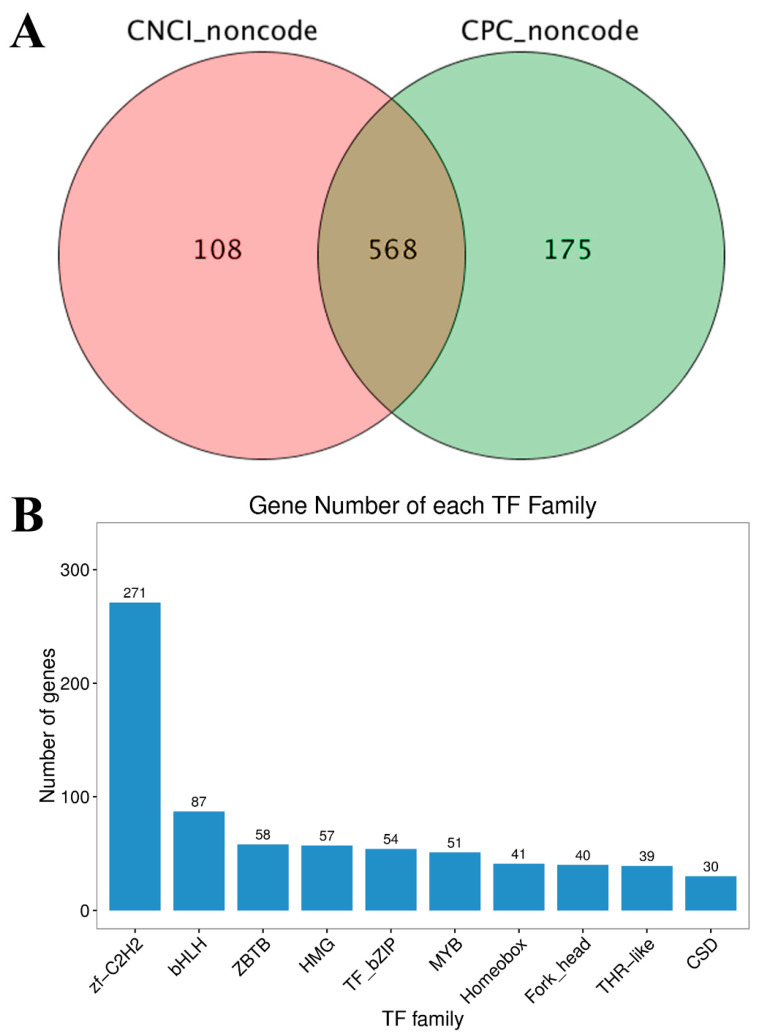
LncRNA prediction and transcription factor classification in *G. cantor*. (**A**) The number of lncRNA transcripts was predicted using CNCI and CPC software. (**B**) The numbers and families of the top 10 transcription factors were predicted using PacBio.

To identify lncRNAs generated from *G. cantor* transcripts, full-length transcriptional sequences not annotated to the four major databases were analyzed. The intersection with a total of 568 potential lncRNAs was formed by the results of the CNCI and CPC software (Figure 3A).

Using hmmscan, 56 TF families were found in the comprehensive set of *G. cantor* transcripts, among which the zf-C2H2 family had the most abundant genes, exceeding those of the other families (Figure 3B). Specifically, 271 and 87 upstream and downstream genes are regulated by zf-C2H2 and bHLH, respectively.

### 2.5. Analysis of AS Events

**Figure 4:**
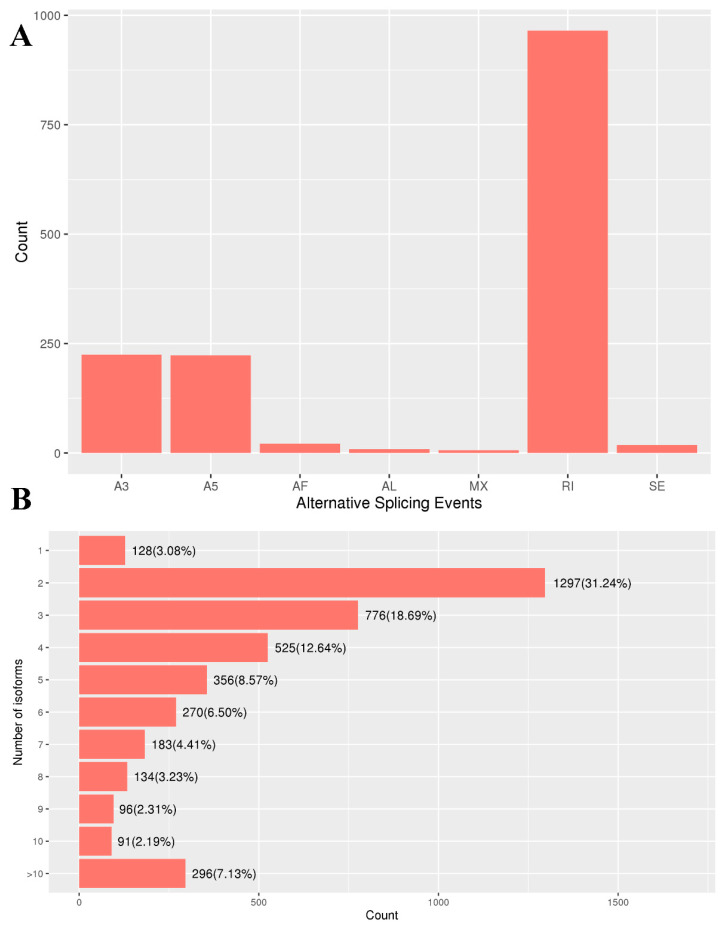
AS events in the full-length transcriptome of *G. cantor*. (**A**) Types of AS events identified in the full-length transcriptome. (**B**) The number of genes containing AS isoforms was counted. The vertical coordinate indicates the number of isoforms, and the horizontal coordinate indicates the number and percentage of genes containing the corresponding number of isoforms.

We determined that alternatively spliced genes produced a total of 1467 alternative splicing (AS) events through seven major types in our sample (Figure 4A), including skipping exon (SE), alternative 5′ splice site (A5), alternative 3′ splice site (A3), alternative first exon (AF), alternative last exon (AL), retained intron (RI), and mutually exclusive exon (MXE) events. RI events accounted for 965 (65.78%) of all events, whereas MXE and AL events accounted for 6 (0.41%) and 9 (0.61%) events, respectively. In terms of quantity, the isoforms were classified into eleven groups: 2 or 3 isoforms after splicing constituted the main group, whereas the two groups with the lowest number of genes corresponded to 9 and 10 isoforms, respectively (Figure 4B).

### 2.6. Potential Functional Analysis of Isoforms with AS Events

**Figure 5:**
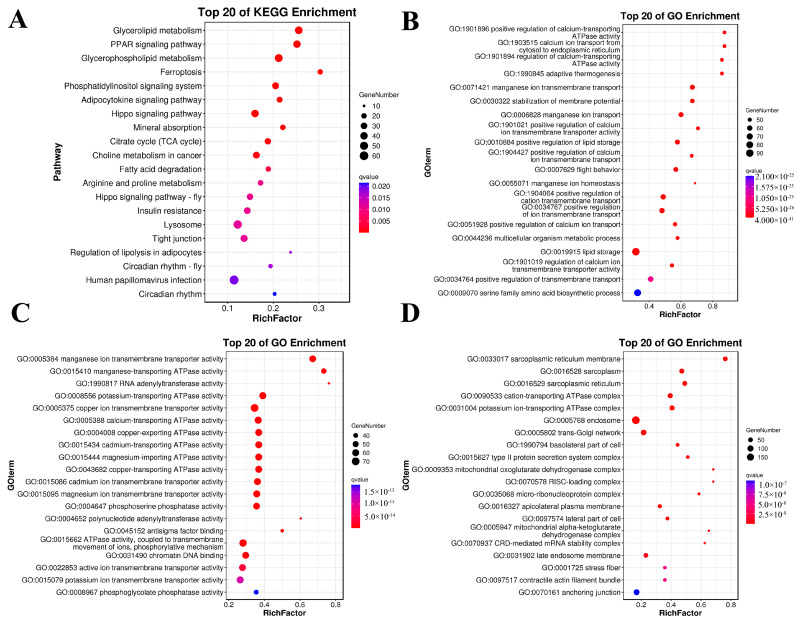
GO and KEGG pathway functional enrichment analysis of isoform AS events. The horizontal coordinate is the Rich factor, which is the ratio of the number of isoforms in the metabolic pathway or GO term to the number of corresponding background isoforms, with larger values indicating greater enrichment. The vertical coordinate indicates the metabolic pathway or GO term with the highest level of enrichment. The color of the dot represents the q value, which is the *p* value after checking. The red color represents a lower value, and a lower value indicates more significant enrichment. The size of the dots represents the number of genes in the pathway. (**A**) Bubble plot of the top 20 enriched KEGG pathways. (**B**) Bubble plot of enriched BPs according to GO terms. (**C**) Bubble plot of enriched MFs of GO terms. (**D**) Bubble plot of enriched CCs of GO terms.

To probe the transcripts after AS that strongly influence the growth and development of *G. cantor*, KEGG and GO enrichment analyses were conducted. As a result, the pathway enrichment analysis indicated that the isoforms were associated with pathways strongly related to glycerolipid metabolism, the PPAR signaling pathway, and glycerophospholipid metabolism (Figure 5A). The enriched GO terms were divided into three categories: biological process (BP), molecular function (MF), and cellular component (CC). The isoforms significantly participated in the positive regulation of calcium-transporting ATPase activity in BP (Figure 5B). Similarly, the transcripts associated with MF were mostly involved in manganese ion transmembrane transporter activity (Figure 5C). Furthermore, splicing isoforms from CCs were enriched predominantly in the sarcoplasmic reticulum membrane (Figure 5D).

### 2.7. GK and GPDH in G. cantor

**Figure 6:**
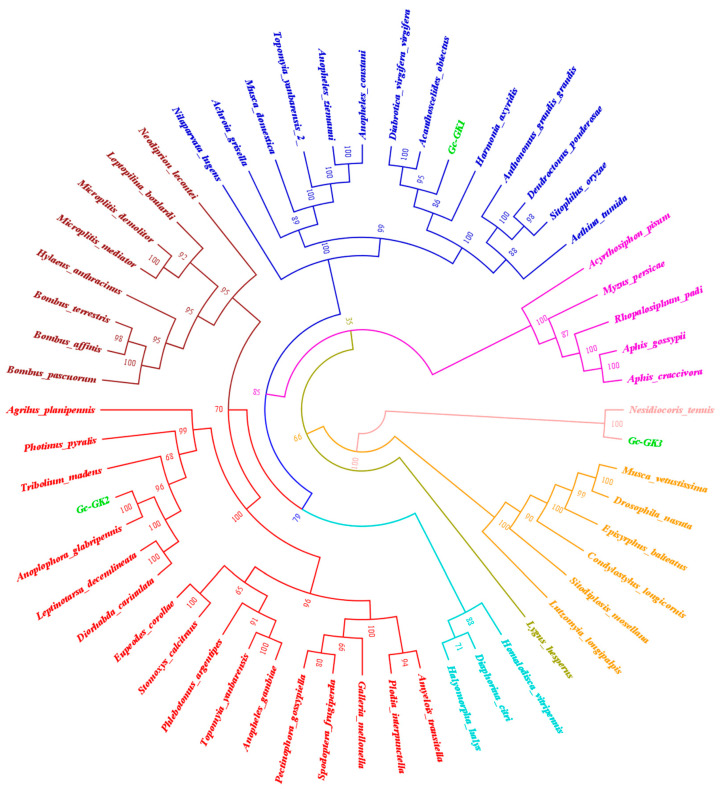
Neighbor-joining (NJ) phylogenetic tree of GK genes from *G. cantor* and other insect species.

**Figure 7:**
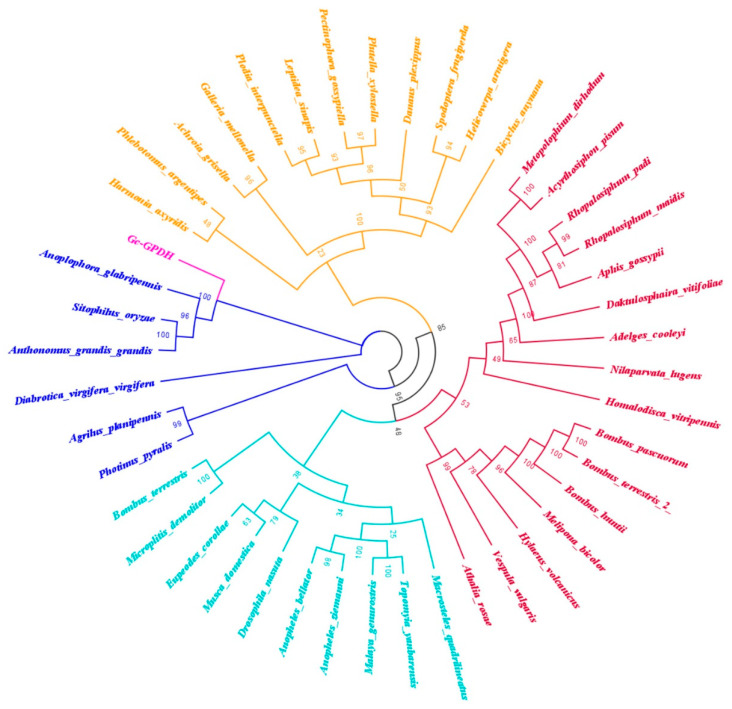
Neighbor-joining (NJ) phylogenetic tree of the GPDH gene from *G. cantor* and other insect species.

Three GK genes and one GPDH gene were identified. A phylogenetic tree confirmed that these GK genes were distributed across different branches and that GcGK2 had the highest homology to *A. glabripennis*. GcGK1 was most similar in origin to Coleoptera insects; however, GcGK3 was clustered into the glycerol kinase gene clan of Hemiptera Pentatomidae (Figure 6). From the perspective of evolutionary kinship, phylogenetic analysis revealed that GcGPDH shares the same ancestor with other Coleoptera species, including *A. glabripennis* (Figure 7).

### 2.8. Different Developmental Stage Analysis of GK and GPDH Expression

**Figure 8:**
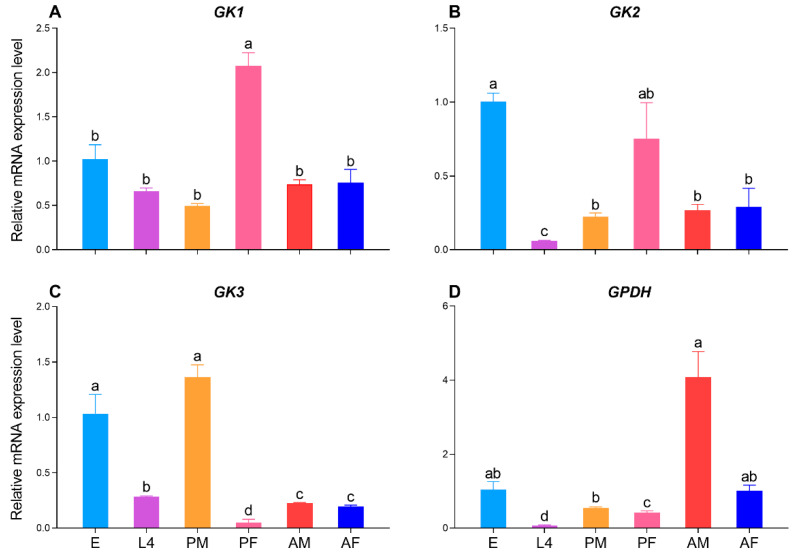
Relative expression levels of the GcGK and GcGPDH genes in different development stages of *G. cantor*. (**A**) GK1; (**B**) GK2; (**C**) GK3; (**D**) GPDH. Note: E: egg, L4: fourth-instar larvae, PM: male pupae, PF: female pupae, AM: male adult, AF: female adult. Significant differences in relative expression levels at different developmental stages are indicated by distinct letters, *p* < 0.05.

For GcGK1, the qRT‒PCR results implied that the relative expression level of female pupae was significantly greater than that at the other stages, while there was no significant difference in the remaining stages (Figure 8A). The egg stage presented the richest transcriptional abundance, whereas the larva of the fourth-instar stage presented the lowest transcriptional abundance, similar to GcGK2 (Figure 8B). Interestingly, in contrast with that in GcGK1, the expression level of the GcGK3 gene in female pupae was highest among all developmental stages, but in male pupae, the highest expression level was detected (Figure 8A,C). The expression level of GcGPDH in male adults was significantly greater than that in the other stages (Figure 8D).

## 3. Discussion

This is the first report of a full-length transcriptome database for *G. cantor*, which provides valuable information from a genetic database of longhorn beetles. The longhorn beetle family comprises approximately 25,000 species that occur throughout the world. However, transcriptome- or genome-related studies are restricted to some species with significant economic losses, e.g., *Monochamus alternatus* [22], *M. saltuarius* [23], *A. glabripennis* [24], and others. Most of these longhorn beetles have 1~2 years for a generation, which may cause difficulties in some aspects of their research. To our knowledge, *G. cantor* has been comprehensively studied as a longhorn beetle model because of its relatively short life history and ability to breed in the laboratory [25]. Together with the previously published antenna and leg transcriptomes [20,21], these data could further contribute to functional and regulatory studies in *G. cantor*.

The results for the AS, lncRNAs, and TFs of *G. cantor* are informative since they are closely involved in development regulation, environmental adaptation, sex differentiation, the innate immune response, and insecticide resistance in insects [26,27,28,29,30,31,32]. AS events are considered a universal feature of eukaryotes and may directly affect the environmental stress adaptation of Coleoptera insects [16]. In *G. cantor*, the diversity of proteins involved in post-transcriptional modifications is principally ensured by the splicing patterns of RIs A3 and A5. Surprisingly, after searching in the results database, we found that GK genes have RI and A3 events. Calcium ions have been proven to be messengers that mediate cold sensing in insect tissues during rapid cold hardening (RCH) [33]. Here, AS event isoforms were enriched not only in glycerolipid and glycerophospholipid metabolism but also in calcium-transporting ATPases, which may cooperate with GK and GPDH in temperature adaptation regulation.

Glycerol has been confirmed to be the main protective agent for several species of insects to resist cold during the overwintering stage [34,35]. The decomposition of glycerol is first catalyzed by GK to form α-glycerol phosphate, which is oxidized and dehydrogenated to generate dihydroxyacetone phosphate through GPDH, the reversibility of which is also the GK/GPDH pathway for synthesis [11]. In *Spodoptera exigua*, GK and GPDH accumulate glycerol, which was identified from overwintering-related genes in larvae [36]. Therefore, GPDH and GK play a role in glycerolipid and glycerophospholipid metabolism since they are conducive to discerning the mechanism of cold resistance adaptability, especially during overwintering or cold hardiness [37,38]. Like *A. glabripennis*, three GPDH genes and one GPDH gene were identified from *G. cantor*, which may also be involved in freeze-proofing and cold resistance physiology strategies [39].

GcGKs were scattered to different genera, particularly when GK3 was on the same branch, as the pest *Nesidiocoris tenuis* was geared toward Miridae. For many metamorphotic insects, the mRNA level of GK is typically increased in the late stage of larvae or adult stages [36]. Our subsequent qRT‒PCR results revealed that GcGK1 and GcGK2 were likely to exert a synergistic effect on female pupae. Conversely, compared with GcGK3, GcGK3 surprisingly occupied a dominant position in male pupae. In summary, the expression patterns of *Gc*GKs differed between male and female pupae. Coincidentally, significant differences in cold adaptability were also detected between male and female pupae (Table 1), indicating that they are likely responsible for the difference in cold adaptability. Moreover, AS events were also detected in GcGK1, whose possible regulation of glycerol biosynthesis may need further evaluation. GPDH is relatively conserved since only one GPDH gene was traced in the transcriptome of other species (Coleoptera, Hymenoptera, and Lepidoptera) [40,41]. Moreover, phylogenetic analysis of GPDH revealed that *G. cantor* shares remarkable homology with other coleopteran insects. Consistent with other insects, our results revealed that GcGPDH functions mainly during the adult stage rather than the larval and pupal stages.

Green control measures, which are inspired by genetics, are still insufficient for *G. cantor* [18]. Owing to the special role of glycerol in insect temperature adaptation, genes for synthesizing glycerol can be used as targets for green pest management [42], which needs further testing via RNA interference. Thus, an in-depth understanding of GcGKs and GcGPDH would not only help to explore the glycerol metabolic mechanism but also prevent the resistance of longhorn beetles to dsRNA pesticides.

## 4. Materials and Methods

### 4.1. Insect Rearing and Sample Preparation

The primary population of *G. cantor* was collected from DaMing Mountain (23°24′ N,108°20′ E) in Nanning, Guangxi Province. They were reared with fresh branches and a few leaves of kapok under conditions of 25 ± 1 °C, 75 ± 5% relative humidity (RH), and a photoperiod of 14 h light and 10 h dark [25]. Data on the distinct developmental stages, adult tissues, and sexes were collected for transcriptome sequencing. Specifically, heads (excluding antennae), thoraxes, wings, abdomens, tarsus, and antennae were dissected from 40 male adults and 40 female adults, which were randomly selected. Next, we prepared eggs (n = 200), 4-instar larvae (n = 3), 6 pupae (F:M = 1:1), and 6 adults (F:M = 1:1). The samples were transferred into cryogenic vials and immediately immersed in liquid nitrogen, after which they were stored at −80 °C until use.

### 4.2. Determination of the Supercooling and Freezing Points

The supercooling and freezing points of mature larvae (4th instar larvae), female pupae, male pupae, male adults, and female adults were measured using an intelligent insect supercooling point tester (SUN-V, Beijing, China) according to the operating manual. At least 30 individuals were tested for each development group.

### 4.3. RNA Extraction, Library Construction and SMRT Sequencing

RNAiso Plus (Takara, Beijing, China) was used to extract total RNA from equal amounts of mixed samples, which were mentioned earlier, according to the manufacturer’s specifications. The integrity of the RNA was determined using an Agilent 2100 Bioanalyzer and agarose gel electrophoresis. The RNA concentration was subsequently measured with a NanoPhotometer (IMPLEN, Munich, Germany). mRNA was enriched with oligo (dT) and magnetic beads, and reverse transcribed into cDNA using the Clontech SMARTer PCR cDNA Synthesis Kit (Clontech, Mountain View, CA, USA). The optimized cycle number was used to generate double-stranded cDNA. Then, the PCR amplification products were purified using AMPure PB Bead. In addition, >5 kb size selection was performed using the BluePippinTM size-selection system, and the fragments were mixed equally with the non-size-selected cDNA. cDNAs were subjected to DNA damage repair, end repair, and linkage with sequencing adapters that were combined with the SMRTbell library. The SMRTbell template was annealed to the sequencing primer, bound to polymerase, and sequenced on the PacBio Sequel II platform by Gene Denovo Biotechnology Co. (Guangzhou, China).

### 4.4. PacBio Data Processing and Annotation Analysis

The raw polymerase reads generated from Sequel II were analyzed by using SMRT Link V8.0.0 [43]. High-quality circular consensus sequences (CCSs) were obtained from subread BAM files. On the basis of whether the CCS reads all contained a 5′ primer, a 3′ primer, and polyA structures, they were classified as either full-length reads or non-full-length reads. The primers, polyA structures, and artificial concatemer consequences were subsequently removed to obtain full-length nonchimeric (FLNC) reads, which were subsequently clustered to generate the entire isoform. Like with the FLNC reads, minimap2 was used for hierarchical clustering to obtain consistent sequences (unpolished consensus isoforms). The Quiver algorithm was subsequently used to further correct the consistency sequence. The high-quality isoforms (prediction accuracy ≥ 0.99) were used to perform sequence analyses.

To annotate the isoforms, the isoforms were analyzed against the NCBI nonredundant protein (Nr) database (http://www.ncbi.nlm.nih.gov), the Swiss-Prot protein database (http://www.expasy.ch/sprot (accessed on 8 September 2021), the Kyoto Encyclopedia of Genes and Genomes (KEGG) database (accessed on 8 September 2021), and the COG/KOG database (http://www.ncbi.nlm.nih.gov/COG (accessed on 8 September 2021) with the BLASTx program (http://www.ncbi.nlm.nih.gov/BLAST/ accessed on 8 September 2021) at an E-value threshold of 1e^−5^ to evaluate sequence similarity with the genes of other species. The Nr protein database was compared with the assembled isoform sequence. The sequence with the best matching result (the lowest E value) of each isoform in the Nr database was taken as the corresponding homologous sequence, and the number of homologous sequences of each species was counted. Blast2GO (version 6.0) software [44] was used to analyze the gene ontology (GO) annotations, and the results of homologous isomer Nr annotation were analyzed. KEGG is a database for systematic analysis of the metabolic pathways and product functions of gene products in cells. Using this database, we further studied the complex biological behavior of genes and isoform pathway annotations through functional information.

### 4.5. Prediction of Long Noncoding RNAs and Transcription Factors (TFs)

CNCI (version 2) software [45] and CPC software (Linux (Intel) version) [46] were used to assess the protein-coding potential of full-length transcript sequences without annotations by default parameters for potential lncRNAs. Both software programs predict the result of “noncoding” and select the intersection of two results as the final result. For invertebrates, protein-coding sequences of isoforms were aligned via hmmscan to the Animal TFdb (http://bioinfo.life.hust.edu.cn/AnimalTFDB4/#/ (accessed on 10 September 2021) to predict TF families.

### 4.6. Alternative Splicing Detection

To explore the AS events of transcript isoforms, the Coding GENome reconstruction tool (Cogent, Washington, DC, USA) [47] was first used to assemble the coding sequences, and the assembled sequences were used as the reference genome. Afterward, SUPPA software (version 2) [48] was used to analyze AS events.

### 4.7. GO and KEGG Enrichment Pathway Analysis of AS Isoforms

To explore the correlations among the isoforms that experienced AS events and their biological functions and processes in detail, we used the isoforms with AS events as candidate gene sets and used all of the isoforms as background gene sets for GO and KEGG enrichment analysis.

### 4.8. Identification and Phylogenetic Analysis of GK and GPDH

Putative glycerol kinase (GK) and glycerol-3-phosphate dehydrogenase (GPDH) isoforms were first screened with the *G. cantor* all isoforms annotation table, where we drew the nucleotide sequences originating from transcriptome isoform FASTA style files. The open reading frames (ORFs) of possibly encoding isoforms were sought by the ORF finder (http://www.ncbi.nlm.nih.gov/gorf/gorf.html (10 November 2023). The new candidates were determined via sequence alignment via DNAMAN and NCBI BLAST (https://blast.ncbi.nlm.nih.gov/Blast.cgi (10 November 2023) for further validation.

The *Gc*GK and *Gc*GPDH genes, as well as their homologous sequences in other species, were downloaded from the NCBI database (accession numbers in the Supporting Information). We constructed phylogenetic trees to explore evolutionary laws by applying MEGA 7.0 software, and the neighbor-joining method with 1000 bootstrap replications was utilized [49].

### 4.9. Relative Expression Analysis of GK and GPDH

First-strand cDNA was synthesized with a Prime Script RT Reagent Kit with a gDNA Eraser (Takara, China). Afterward, we carried out a quantitative real-time polymerase chain reaction (qRT‒PCR) with TB Green Premix Ex TaqII (Takara, China), which was run on a LightCycler 480 system following the manufacturer’s instructions. GcEF1A1 (GenBank: MW462104) and GcRPL36 (GenBank: MW462098) were used as internal reference genes [50]. Appropriate primers (Table S1) for qRT‒PCR were designed using NCBI tools (https://www.ncbi.nlm.nih.gov/tools/primer-blast/ (13 November 2023). Three biological replicates and three technical replicates were performed for each experiment. The 2^−ΔΔCt^ method was used to calculate the relative expression levels of genes [51]. All of the data were processed with SPSS 27.0 (IBM) and GraphPad Prism 8 (America). For the parametric test, the homogeneity of variance followed Tukey’s HSD test (*p* < 0.05); otherwise, the nonparametric test was used with the Kruskal–Wallis test (*p* < 0.05).

## 5. Conclusions

Overall, a comprehensive set of isoforms will improve fundamental projects revealing the causes of behavior in *G. cantor*. Underlying AS events that have been extended for KEGG enrichment analysis were forecasted to seek key elements in elucidating the complexity of glycerol synthesis. Identification and preliminary characterization of GcGKs and GcGPDH suggested a sex difference in GK1 and GK3, which broadens the horizons of the cryoprotectant metabolic system. This research provides a point of departure for elucidating the overwintering mechanism and lays a firm foundation for understanding the role of cold-resistant substances in temperature adaptation from a microscopic perspective.

## Figures and Tables

**Table 1:** Supercooling points and freezing points at different stages of *G. cantor* development.

Insect Stage	Supercooling Point			Freezing Point		
	Maximum	Minimum	Mean ± SE	Maximum	Minimum	Mean ± SE
Mature larvae	−11.26	−18.85	−14.39 ± 0.68 c	−2.63	−16.74	−9.20 ± 1.14 c
Female pupae	−19.23	−23.03	−22.16 ± 0.35 a	−6.90	−18.61	−10.04 ± 0.97 bc
Male pupae	−15.65	−23.22	−20.15 ± 0.83 b	−7.66	−18.91	−13.95 ± 1.16 a
Female adult	−11.74	−18.12	−14.96 ± 0.59 c	−8.46	−17.97	−13.38 ± 0.80 a
Male adult	−11.49	−17.21	−17.21 ± 0.53 c	−7.78	−16.22	−12.50 ± 0.77 ab

Note: Data in the table are the means ± SEs, and different letters in the same column indicate significant differences based on DMRT (*p* < 0.05).

**Table 2:** Overview of the full-length transcriptome of *G. cantor.*

Category	Number
Total base (bp)	22,459,530,141
Subreads number	9,716,761
Subreads average length	2311
Circular consensus sequences (CCS)	363,474
CCS read length (mean)	2780
High-quality isoforms	26,275
low-quality isoforms	1136
High-quality nonredundant isoforms	24,476
Average of high-quality nonredundant transcripts	2686

High-quality sequences are sequences with an accuracy greater than 99%.

## Data Availability

Data is contained within the article and Supplementary Materials.

## Supplementary Materials

The following supporting information can be downloaded at: https://www.mdpi.com/article/10.3390/ijms252111834/s1.
