# Supplementary material for: Transcriptome and Expression Analysis of Glycerol Biosynthesis-Related Genes in Glenea cantor Fabricius (Cerambycidae: Lamiinae)
Source: Int J Mol Sci. 2024 Nov 4;25(21):11834. doi: 10.3390/ijms252111834 (PMC11545850; doi:10.3390/ijms252111834)
Supplement: Supplementary file 1 [file ijms-25-11834-s001.zip › ijms-3252852-supplementary.pdf]

Supporting Information

Table S1 Primers for qRT-PCR in *G. cantor*

Primers	Primer Sequence (5'-3')
GcGK1-F	AGTGGAAGATGGCGGTTCAG
GcGK1-R	GGTACGCTGGCCAATAACCT
GcGK2-F	ACAGTGGCCTACAAGTTGGG
GcGK2-R	CAACCAGTTCAATGCAGCCC
GcGK3-F	AGAGTGGATGCTGAACACGG
GcGK3-R	ACCCACTGAACGCAGGTATG
GcGPDH-F	CTCCCACATCATCACCCGTC
GcGPDH-R	TTGCAGCCTATGGTGGTCTC
GcEAFA1-F	TGGCGATGCTGCCATTAT
GcEAFA1-R	GGACAGCGAAACGTCCTAAT
GcRPL36-F	GAAATTCGTGCGAGACCTCATC
GcRPL36-R	GGCGGCGCTTAAGGAATTTA

Table S2 The top10 pathway enrichment of *G. cantor*

	Pathway	All genes with pathway annotation (9492)	Pathway ID
1	Metabolic pathways	2679 (28.22%)	ko01100
2	Pathways in cancer	660 (6.95%)	ko05200
3	Human papillomavirus infection	563 (5.93%)	ko05165
4	Alzheimer disease	542 (5.71%)	ko05010
5	PI3K-Akt signaling pathway	445 (4.69%)	ko04151
6	Lysosome	443 (4.67%)	ko04142
7	Protein processing in endoplasmic reticulum	438 (4.61%)	ko04141
8	Huntington disease	413 (4.35%)	ko05016
9	Hypertrophic cardiomyopathy (HCM)	379 (3.99%)	ko05410
10	Proteoglycans in cancer	371 (3.91%)	ko05205

The nucleotide sequence of 3 and 1 GK and GPDH in *G. cantor*

>GcGK1

ATGGAAACAAGTTCGAGTTCAGAAAAGCCTTTAATTGGAGTGATAGATGCCGGAACAA

GGACGGTAAAGTTTTGCGTTTTTCGAAGTCAAGAAACCAAAGAAATAGCAGAACACA

CCATCGATATTAGCACATATACTCCAAAAGAAGGATGGTCGGAACAGGATCCAAAGGA

AATTTTAGATGCTGTGAAAAGTTGTATGGAAAAAGTAGTTAAGTGTTTAGGATCAGAAA

GTGAAAAAAATTCGGGCGATAGGTATAACAAACCAAAGGGAAACGACTATACTGTGGGA

CAAAACGACCGGCGAACCTTTGTACAATGCCTTAGTGTGGAACGACATAAGGACAGAT

TCCACGGTGGACATAATTCTGGCCAAAGTACCAGAAAACAATACAAACTATTACAAAC

CAATATGTGGTCTTCCGATATCTCCGTACTTTAGTGCTTTTAAGGTCAAGTGGCTTATGC

ATTACGTTTCTGACGTTAAAAAGGCGGTCACTGCCAAAAAGTGCCTTTTTGGAACTGT

CGACACATGGATATTGTGGAATTTGACGGGTGGGGTGAATGGCGGTTTACACTTAACG

GACGTGACTAACGCGTCTCGCACATTCCTTATGAACATAGAATTACTGAACTGGGATCC

GGTACTGTTGCACACCTTTAAGATCCCTCCGGAGATCCTGCCAGAGATCCGGAGCAGC

TCAGAAATTTATGGTCGCATCGCCAAGGGCTTAACTTTGGAAGACGTGCCTATATCCGG

GATCTTAGGCAACCAACAGGCGTCCCTGCTAGGTCAAGGCTGCATGAGGGAAGGTGA

GGCCAAAAACACGTACAGGAGCGGATGTTTCCTACTTTACAACACCGGCAATTCGAAA

GTACAATCGACTCATGGGTTAGTGACGACGGTGGCTTACAAGTTTGGGGACAAACCGG

CGGTTTTCGCGTTGGAAGGAAGTGTGGCGGTAGCTGGGGCGGCCATGAAGTGGTTGA

GGGACAACATGGGTTTCCTGAACGATGTGCAGAGGGACACGGAATCTTTAGCTCAGGA

AGTGTTCAATACCGGCGATGTATACTTTGTACCTGCCTTTAAGGGCCTCTATGCTCCATA

TTGGAGGAAAGACGCAAGAGGGATCATTTGCGGGCTGACGGCGTTTTCCACGAAACA

GCACATAGTTAGAGCCGCTTTGGAAGCTGTCTGCTTCCAGACGAGGGACATCTTAGAG

GCCATGAACAAGGACTGCGGCATACCGCTTTCCAAACTGCACGTCGACGGTAAAATGA

CGCAGAACAGCCTCCTCATGCAGCTGCAGGCGGATATCAGCGGCATACCCGTTATACG

GGCGCAATCGCAGGACATCACCGCCTTGGGGATGGCCATGGCGGCGGGATCGGCGCA

GGGGATCGACGTGTGGGACCTCGAAGCCGAGGACCGTCAGCTGGTGCCTAGCGATAC

ATTCCTTCCCACGTCGACGGAGGACGAGAGAGACGCGAGGTACACGAAGTGGAAGAT

GGCGGTTCAGAGGTCGCTGGGATGGTCGGTAGCGAAGAAGTCAGAAGCTATGACAGA

GGAGCGGTACAGGTTATTGGCCAGCGTACCGGGAAGCCTGTACATTATGTCGAGCTTC

GGCTTGATAGCCCTCTCGGAGTATCTAACGAAAGCCGTATAA

> GcGK2

ATGGGACCCCTGGTAGGAGCTATCGACGAGGGCACGTCAAGTGCGAGATTTATTCTATT

TGAGGCTGGAACTACAAATGTGGTGGCTTCACATCAACAGGAATTATCGCAAATTTACC

CGCAGGAAGGATGGGTTGAGCAGGACCCCATGGAAATTTTGAGAGTCGTAAGAACTT

GCATTGAGAATGCCATCGATAAGCTCATAGCCTCAGGAGGTAGTGTGAATGATATCGTA

GCAGTAGGTGTAACAAATCAAAGGGAGTCTACTATAGTTTGGGATAAAACTACCGGAG

AGCCTTTGTACAACTCCATTGTATGGTTAGACGTGAGGACTTCGTCGACTGTTGATCAA

CTTCTTGATGAGGTGCCGAATAAATCCAGAAATAAAAATTATTTGAAACCCCTCTGCGG

TTTGCCTCTTTCGCCTTATTTTAGCGCAGTTAAGTTGAGGTGGCTTCAAGACAACATCT

CCAAAGTTAAAAAGGCCATGGTAGCCGGAAACTGCTTATTCGGTACCGTAGACAGCTG

GTTGATATGGAACTTGACGGGCGGTGTAAACGGGGGAATTCACATAACCGACGTGTCC

AACGCATCCAGAACCATGTTGATGAACATCGAGACGTTAAAGTGGGACCCCGTTTTGA

TCAATTTCTTCGGTATCCCGAGTTCTGTTCTCCCAACCATTAAATCCAGTTCGGAAGTCT

ATGGAAATATCGACGAGGGTTCGCTTAGGGGAGTTCCGGTTTCCGGATGCCTCGGGGA

CCAGCAGTCTGCTTTGGTAGGACAGCAGTGCCTTAATCGAGGACAGGCTAAAGCGACA

TACGGAACCGGTTGTTTTCTTCTCTACAACACCGGCAATATAAGAGTGCACAGCACCA

AAGGCCTCGTGACAACAGTGGCCTACAAGTTGGGCCCCAAAAGCCCAGCGGTCTACG

CTTTGGAGGGGTCAGTCGCCATCGCAGGGGCTGCATTGAACTGGTTGAGGGACAACCT

ATCGGTTTTGTCAAGTTTCGGGGAAGCCCAAGAAATTTCCGAGAAAGCCGCCAAGATG

CGGGACGGCGAGGTGTACTTTGTGCCGGCTTTCAGCGGTCTTTACGCCCCCTACTGGC

AGCAAGAAGCTAGAGGAGTTATTGTGGGAATCTCGGAGGATACGGAAGCTTGTCACAT

CGTCAGGGCTACATTAGAGGCTGTGTGCTTCCAAGTGAGAGATATCTTAGAAGCCATG

AATAAGGATTACGGTTCCAATCTGACGAGTCTGCAGGTGGACGGCGGCATGACGGCCA

GCTCCCTCCTGATGCAACTGCAGGCGGACTTGGCGGGGGTGACGGTGTTAAAACCGA

CGATGGCGGAGAGCACCGCTCTGGGGGCGGCGATGGTGGCTGGGGCGGCGGTGGGCC

ACTGGAACTTGTCCGGTCCCCCGCTGGATATACCGGCTATAAGATGGTCCCCGAAATTC

ACCGAGGACGAGAGGGACGTCGCGTACTCCAAGTGGAAGATGGCCGTGGAACGGTCC

ATGGGGTGGGTTGTGTAA

> GcGK3

ATGTATAAGTCTGGCGACACTCCTGATTATGTAGCAGCACTAGATATAGGAACAACAAC
TATCAGATGTCAGATAATTAATTCTCGTACTGAAACTGTTGGAGTAGCTTATTCAGCTGT
ACAGTTGCATTATCCCAAACCAGGATATGTGGAAATCATTCCCGACAAACTTTTAGAGG
AAATTATAAAAGTCGTGAAACAGGCAGTAGAAGATGCAAAAATCAGCATTTCCGAGAT
TAAATCAGTTGGAATCTCGACGCAAAGGGCCACATTTATAACGTGGAAAAAGGATTCT
GGGGAATATTTACACAATCTCATAACTTGGAAAGATTTACGAGCTGTGACATTAATCAA
GCAAGTTAACTCTGCGTGGCTTACTAACGTTGTACGTTGGGCAGCATACTTCCTTTATCT
GATATCGAGAAACAAAAGGTATGGAGTGGCAGCAAATTAAAATTCTGCAGCAATCAT
GTGTCCATCAGGCTGCTGTGGGTTCTTCAGAATATTAAAGAAGCTAAAGAGGCGGCGG
AAAAAAACAACCTAATGTTCGGAACTTTAGAGACTTGGTTGATCTACAAGCTTACAGG
AGGAGAAACGTACGTCACGGACATATCCAACGCCAGCGCTACGGGTCTGTACGACCCC
TTTGACCTGGACTGGGGCATTATGACGAATATATTGGGGATACCTAGGAATATTTTTCCT
AAAATTGTTAACAACGACTATGAATTCGGGAGCATAAAAACCCATTATTTTGGGATGCC
CATTAAAATCGGATGTGTGATGGCAGATCAGTCGGCATCCATGTTTGGTTCTTGTTCTTT
CTACAATCATGACATCAAAGTTACCATGGGTACGGGGGCATTTCTCGACGTAAACACGT
CCACGAACATCCATCCCAGTTTAAATGGAATGTACCCTTTAATAGGGTGGAAATTAAAG
GACGAGATTGTTTATTTGACAGAATCGTCCTGTCTGGATGCGGGGTCCTTGATAGAGTG
GATGCTGAACACGGGCTTCATTAATGAACCTACAGAAACGGCCGATATGGCTCTTAAA
ATTAACGACACTGATGGAGTTTTCTTCATACCTGCGTTCAGTGGGTTAGGGCCGCCTAT
TAACAACGAAAAAGCAGCAACTGGTTTCATCGGCATAAAACCAACTACTTCTAGAGAA
CATCTGGTGAGGGCAGTATTGGAAAGCATAGTTTTTAGAGTCGCCCTCGCCTACGATTT
ATTAAAGGCGGAAAGGTGTGAAAACTACAAGACCATTAGAATAGACGGAGGAGTGTC
GAGAAATAATTTTATTTGCCAGATGCTCGCAGATCTAACGGGTTTAGCGGTGGAGAGGC
AAAGTTCGGAAATGTCAGTGTTAGGTGTTGCATTTTTAGCAGGAATAACTTGCGGTATA
TGGAGCGATAAGGAACAATTGAAAGAGTTCCATGAGACGGAAAAAGTATTTACACCAT
GCACATCAGAGGAATATAAAAATGCATGTAGAAGGAATTTAAGTGAATGGATGGTTGC
GGCTTATAGATTTAAGTCGTGGTACAAAGAAAATTAA

> GcGPDH

ATGATTGGTAATACAGTTTTTAACCAAAATTCTATTGGCCGGGTCATAGTTGTTTTTAAA

CGACGTCGACTTTGTGCGATTCCGATATTAACTTGTAGTTCGTGTTCGCGTGAAATAGTT

GTTAATCAGTCGCCGCAGTTGTCGGTTCGTCCTTATCGTGGTTATTCCACATCAAAATTT

TTAGTTAATTACACAACCAAAATGGCTTGTACAAAACCAAAAAGGGTTTGCATTGTAG

GTAGTGGCAACTGGGGCTCTGCAATTGCAAAGATCGTTGGCAAAAATGCTGTGCAACT

TTCTAATTTTGATGACAAGGTTACTATGTATGTTTATGAAGAAATAATAAATGGAAAAAA

ATTGACTGAAATTATAAATGACACCCATGAAAATGTGAAATATTTACCAGGGCATAAATT

GCCTCCCAATGTGGTTGCTGTACCCGATGTTGTAGATGCAGCGAAAGATGCAGATATTC

TTATATTTGTTGTGCCCCATCAGTTTATAAGAACATTGTGCTCAACTTTATTAGGAAAAA

TTAAACCAACTGCAATTGCTTTGTCATTGATCAAGGGATTCGATCGCGCAGAAGGTGG

AGGCATAGACCTAATCTCCCACATCATCACCCGTCACCTGAAAATCCCGTGCGCCGTGC

TGATGGGCGCAAATCTAGCTGGCGAGGTGGCCGACGAGAAATTCTGCGAGACCACCAT

AGGCTGCAAAGACATCCAGCTGGGCCCCGTGCTCAGAGACATCATCCAGACCAACTAC

TTCAGGGTGGTTGTCGTCGACGACGAGGATACCGTCGAAGTGTGCGGAGCTCTTAAGA

ATATCGTAGCCTGCGGAGCCGGCTTCGTCGACGGTTTAGGCTTGGGGGACAACACCAA

GGCCGCCGTCATCCGACTAGGATTGATGGAGATGGTGAAATTTGTGGACGTATTCTATC

CTGGTGGCAAGCTGGCCACTTTCTTTGAGAGCTGCGGCGTGGCTGATCTCATAACCAC

CTGCTACGGAGGTCGCAACAGGAAAGTCAGCGAGGCATTCGTGAAGACCGGCAAGTC

GATAAAGGAGCTGGAGGATGAGCTGTTGAATGGCCAAAAGCTACAAGGTCCCTTCACC

GCCGAAGAGGTGAATTACATGTTGAGGAACAGGGACATGGAGGACAGGTTTCCGCTG

TTCACGGCCATCCACAAGATTTGTACGGGGCAGTTGCCAGCGCAGGAATTTATAGATTG

TATCAGGAACCACCCTGAGCACATGGTTAGAGGTGCATCCAGTTAA

The accession number for various species in GK gene phylogenetic tree.

species	accession number
Rhopalosiphum padi	XP_060844228.1
Aphis gossypii	XP_027851749.1

Nesidiocoris tenuis	BES90209.1
Homalodisca vitripennis	XP_046676071.1
Nilaparvata lugens	XP_039281267.1
Aphis craccivora	KAF0772382.1
Acyrthosiphon pisum	XP_016660307.1
Diaphorina citri	XP_008469116.1
Halyomorpha halys	XP_024216973.1
Myzus persicae	XP_022172698.1
Lygus Hesperus	JAG35158.1
Amyelois transitella	XP_060809509.1
Achroia grisella	XP_059045853.1
Plodia interpunctella	XP_053624052.1
Galleria mellonella	XP_052752500.1
Spodoptera frugiperda	XP_035429035.1
Pectinophora gossypiella	XP_049886227.1
Bombus pascuorum	XP_060811859.1
Microplitis mediator	XP_057321465.1
Hylaeus anthracinus	XP_054007974.1
Microplitis demolitor	XP_008546991.1
Leptopilina boulardi	XP_051174454.1
Bombus affinis	XP_050578300.1
Bombus terrestris	XP_003394582.1

Neodiprion lecontei	XP_015516193.1
Diorhabda carinulata	XP_057672179.1
Diabrotica virgifera virgifera	XP_028134815.1
Anthonomus grandis grandis	XP_050312360.1
Aethina tumida	XP_019871562.1
Acanthoscelides obtectus	CAK1647795.1
Dendroctonus ponderosae	XP_048525395.1
Harmonia axyridis	XP_045478379.1
Tribolium madens	XP_044268892.1
Leptinotarsa decemlineata	XP_023015333.1
Photinus pyralis	XP_031356436.1
Sitophilus oryzae	XP_030767742.1
Agrilus planipennis	XP_025836773.1
Anoplophora glabripennis	XP_018571090.1
Anopheles gambiae	XP_061506539.1
Musca vetustissima	XP_061391482.1
Drosophila nasuta	XP_060655978.1
Phlebotomus argentipes	XP_059614843.1
Stomoxys calcitrans	XP_013097421.2
Musca domestica	XP_011296357.1
Topomyia yanbarensis-1	XP_058826953.1
Topomyia yanbarensis-2	XP_058825349.1

Anopheles ziemanni	XP_058174836.1
Anopheles coustani	XP_058130533.1
Eupeodes corollae	XP_055911455.1
Episyrphus balteatus	XP_055855934.1
Lutzomyia longipalpis	XP_055693793.1
Condylostylus longicornis	XP_055382290.1
Sitodiplosis mosellana	XP_055315939.1

The accession number for various species in GPDH gene phylogenetic tree.

species	accession number
Anthonomus grandis grandis	XP_050297873.1
Harmonia axyridis	XP_045465915.1
Photinus pyralis	XP_031358876.1
Sitophilus oryzae	XP_030754652.1
Agrilus planipennis	XP_025832249.1
Anoplophora glabripennis	XP_018575858.1
Metopolophium dirhodum	XP_060873676.1
Rhopalosiphum padi	XP_060849200.1
Macrosteles quadrilineatus	XP_054281543.1
Daktulosphaira vitifoliae	XP_050520852.1

Adelges cooleyi	XP_050443623.1
Aphis gossypii	XP_050058615.1
Homalodisca vitripennis	XP_046684301.1
Nilaparvata lugens	XP_022196867.1
Acyrthosiphon pisum	XP_008184495.1
[Rhopalosiphum maidis	XP_026820454.1
Achroia grisella	XP_059046124.1
Plodia interpunctella	XP_053603284.1
Bicyclus anynana	XP_023944955.2
Galleria mellonella	XP_026755779.1
Leptidea sinapis	XP_050681388.1
Spodoptera frugiperda	XP_035438355.1
Pectinophora gossypiella	XP_049869964.1
Helicoverpa armigera	XP_049694767.1
Danaus Plexippus	XP_032512602.1
Plutella xylostella	XP_048478972.1
Bombus pascuorum	XP_060813611.1
Hylaeus volcanicus	XP_053986857.1
Microplitis demolitor	XP_053595246.1
Vespula vulgaris	XP_050868177.1
Bombus huntii	XP_050474024.1

Melipona bicolor	KAK1120443.1
Athalia rosae	XP_020710418.2
Bombus terrestris	XP_003397690.1
Bombus terrestris-2	XP_020720012.1
Drosophila nasuta	XP_060654475.1
Phlebotomus argentipes	XP_059610095.1
Musca domestica	XP_011294614.1
Topomyia yanbarensis	XP_058826644.1
Malaya genurostris	XP_058466329.1
Anopheles ziemanni	XP_058176740.1
Anopheles Bellator	XP_058053725.1
Eupeodes corollae	XP_055923687.1

The accession number for nucleotide sequences of GK and GPDH genes

Glycerol kinase 1	PP965667
Glycerol kinase 2	PP965668
Glycerol kinase 3	PP965669
Glycerol-3-phosphate dehydrogenase	PP965670
